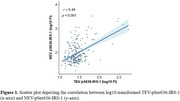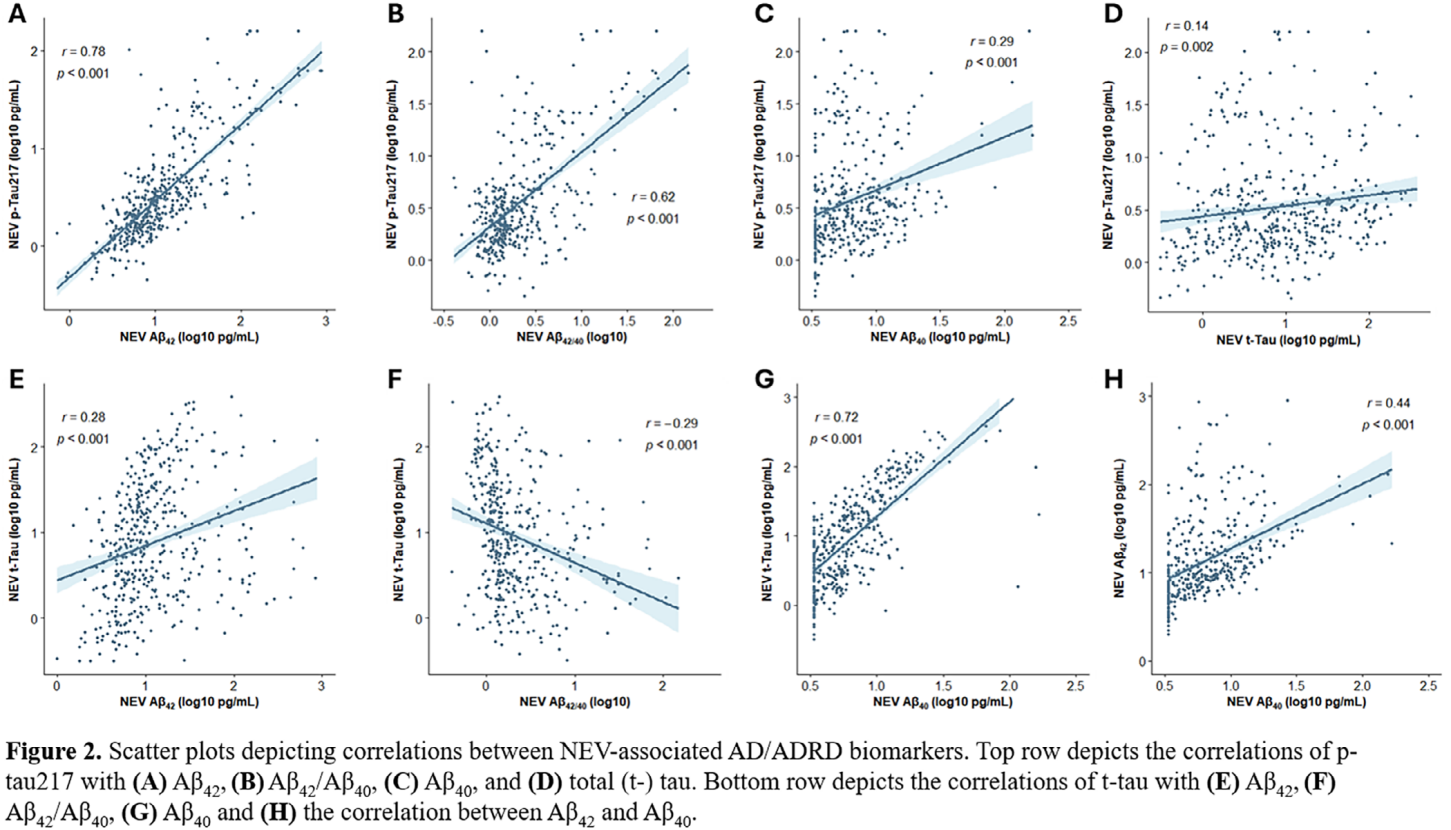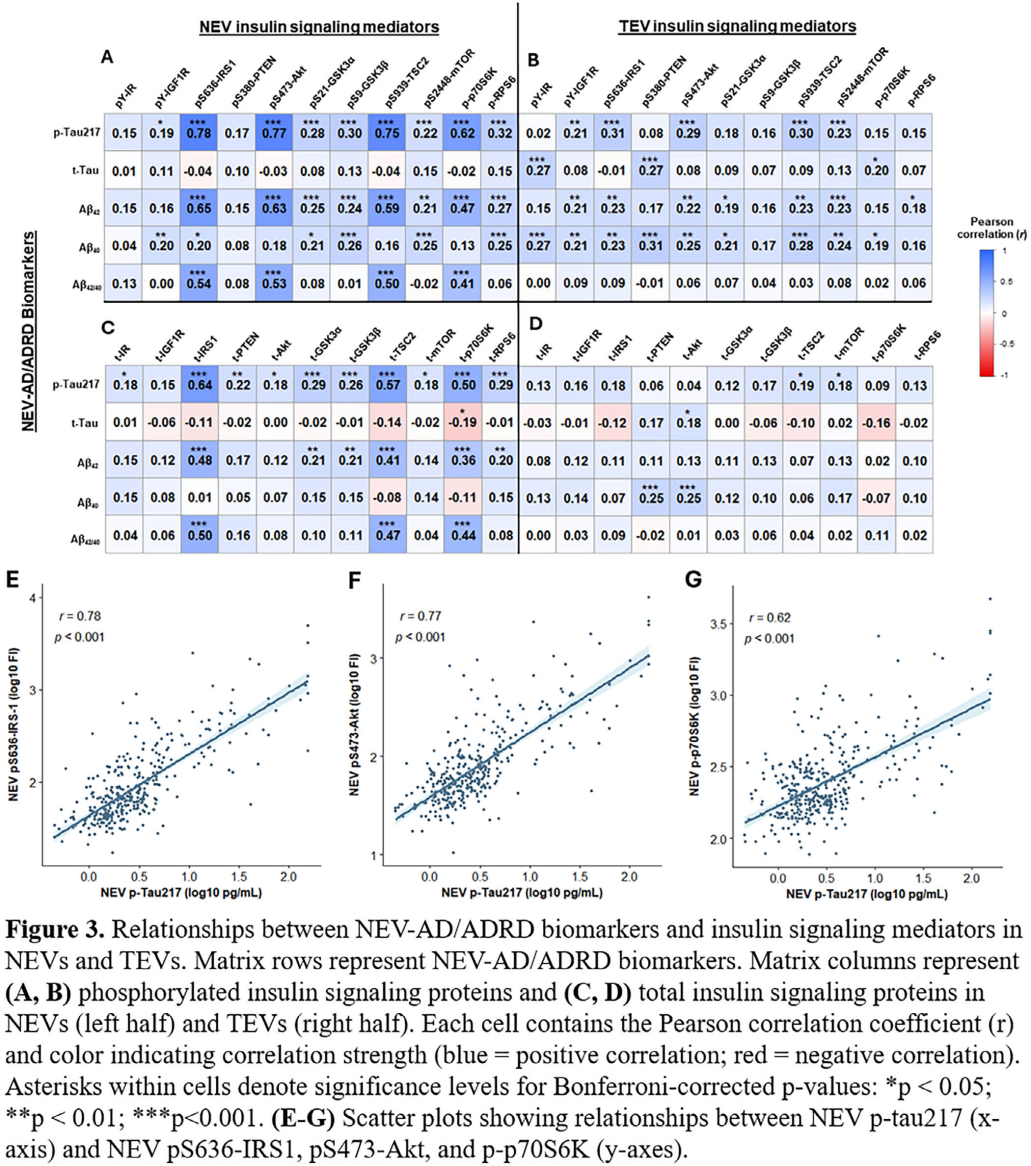# Neuronal extracellular vesicle biomarkers of dysregulated insulin signaling and Alzheimer’s disease are strongly associated in prediabetes and type 2 diabetes

**DOI:** 10.1002/alz70861_108176

**Published:** 2025-12-23

**Authors:** Saswat Sahoo, Darlene Antoine, Maja Mustapic, Tushar Dubey, Alexander Daskalopoulos, Blandine Laferrère, Dana Dabelea, Owen T. Carmichael, Pandora L Wander, Steven Kahn, Vallabh Shah, Marinella Temprosa, José A Luchsinger, Dimitrios Kapogiannis

**Affiliations:** ^1^ Cleveland Clinic Lerner College of Medicine, Cleveland, OH USA; ^2^ National Institute on Aging, Baltimore, MD USA; ^3^ Columbia University Irving Medical Center, New York, NY USA; ^4^ Colorado School of Public Health, Aurora, CO USA; ^5^ Pennington Biomedical Research Center, Baton Rouge, LA USA; ^6^ University of Washington, Seattle, WA USA; ^7^ University of New Mexico, Albuquerque, NM USA; ^8^ George Washington University, Rockville, MD USA

## Abstract

**Background:**

Dysregulated insulin signaling in the IRS‐1/Akt/mTOR pathway underlies insulin resistance in prediabetes (PreD) and type 2 diabetes (T2D) and may contribute to the pathogenesis of Alzheimer’s disease and related dementias (AD/ADRD). However, the relationship between neuronal and systemic insulin signaling abnormalities and their association with AD/ADRD biomarkers in PreD/T2D remains unclear. Plasma‐derived extracellular vesicles (EVs) carry signaling and pathogenic proteins, providing a platform to examine molecular relationships between PreD/T2D and AD/ADRD pathophysiology.

**Method:**

In 456 participants with PreD/T2D from the Diabetes Prevention Program Outcomes Study, we isolated neuronal and total circulating EVs (NEVs and TEVs) from fasting plasma collected at Year 22 follow‐up visits using size‐exclusion chromatography followed by immunocapture (NEVs: anti‐L1CAM/NLGN3/GAP43; TEVs: anti‐CD9/CD63/CD81). NEV‐associated *p* ‐tau217, t‐tau, Aβ_42_, and Aβ_40_ were quantified by SIMOA. Phosphorylated and total levels of IR, IGF1R, IRS‐1, PTEN, Akt, mTOR, p70S6K, GSK3α/β, TSC2, and RPS6 were measured in NEVs and TEVs (reflecting neuronal and systemic insulin signaling, respectively) using Luminex immunoassays. Cross‐sectional biomarker associations were examined using Pearson's correlations with Bonferroni adjustment for multiple testing.

**Result:**

pS636‐IRS‐1, a marker associated with impaired insulin signaling, was moderately correlated between NEVs and TEVs (r=0.49; Figure 1). NEV‐associated AD/ADRD biomarkers were intercorrelated (Figure 2), with strong associations observed between *p* ‐tau217 and Aβ_42_ (r=0.78), *p* ‐tau217 and Aβ_42_/Aβ_40_ (r=0.62), and t‐tau and Aβ_40_ (r=0.72). Accordingly, *p* ‐tau217, Aβ_42_, and Aβ_42_/Aβ_40_ showed moderate‐to‐strong correlations with NEV‐associated phosphoproteins linked to insulin resistance in histopathologic studies (Figure 3A). Among NEV‐associated AD/ADRD biomarkers, *p* ‐tau217 demonstrated the strongest correlations with key NEV‐associated insulin signaling phosphoproteins (pS636‐IRS1: r=0.78; pS473‐Akt: r=0.77; *p* ‐p70S6K: r=0.62; Figures 3E‐G). In contrast, correlations between *p* ‐tau217 and the same phosphoproteins in TEVs were weaker (pS636‐IRS1: r=0.31; pS473‐Akt: r=0.29; *p* ‐p70S6K: r=0.15). Correlations for t‐tau and Aβ_40_ with insulin signaling mediators were weak overall.

**Conclusion:**

In individuals with PreD/T2D, NEV‐associated AD/ADRD biomarkers were more strongly associated with dysregulated insulin signaling mediators in NEVs than those in TEVs. Strong correlations were observed for NEV‐associated pS636‐IRS‐1 and pS473‐Akt with *p* ‐tau217 and Aβ_42_, suggesting a link between dysregulated neuronal insulin signaling and AD/ADRD pathophysiology. Future studies will determine how these relationships relate to metabolic and cognitive measures.